# A Guide to the Clinical Examination of the Blood for Diagnostic Purposes

**Published:** 1897-12

**Authors:** 


A GUIDE TO THE CLINICAL EXAMINATION OF THE BLOOD FOR DIAGNOSTIC PURPOSES. By RICHARD C. CABOT, M. D. With colored plates and engravings. New York: William Wood & Co. 1897.

Through an unavoidable oversight the review of this most excellent treatise has not appeared in the JOURNAL until now, some months after its appearance and hearty reception by the medical press and the profession.

Hematology is practically a new topic, yet there are several very excellent treatises published upon the subject, particularly by German and French writers. The present volume is by far the most exhaustive and reliable of those published in the English language and has attained in a few months a very flattering reputation.

The author claims that an examination of the blood gives evidence similar in kind and not much inferior in value to that obtained by examination of the urine. Both, he claims, gives us (*a*) a ready-made diagnosis in a few cases, (*b*) side lights on a good many obscure conditions, (*c*) and the frequently great assistance of a negative report. It has solved some problems where little was hoped from it and has been disappointing where much was expected—as for instance in rheumatism, furunculosis, uremia and diabetes. In the diagnosis of central pneumonia, deep-seated suppurations, ovarian tumors and in the prognosis of post-scarlatinal nephritis and pneumonia, the examination of the blood has been unexpectedly helpful. The chapters on the methods of clinical examination of the blood consider the counting of the red and white corpuscles, the estimation of the relative volumes of corpuscles and plasma ; of the coloring matter, of the specific gravity, the examination of aired and stained specimens and the bacteriological examination.

For the blood count, the author mentions only the Thoma-Zeiss instrument—it being the most commonly used and the most accurate. v. Fleischl's hemometer is the preferred instrument for determining the amount of hemoglobin, while the Hammerschlag method is given as the simplest and most available method for estimating the specific gravity of the blood. The author almost recommends the specific gravity method for the determination of hemoglobin—it being cheaper, easier, more accurate and equally quick—than the use of the hemometer.

Part II. is devoted to a study of the physiology of the blood and only such portions as are necessary for an understanding of the small group of pathological changes, which can be profitably investigated by clinicians. The morphology of the blood, its coloring matter and its density under physiological conditions are especially noted.

Part III. is devoted to the general pathology of the blood—such as its unequal distribution, its dilution and concentration, anemia and hydremias. The most common causes of secondary anemia are given as infective and febrile diseases ; malignant disease, chronic suppuration ; bad hygiene, pregnancy and lactation ; intestinal parasites, poisons, lead, arsenic, mercury. The chapter on leucocytosis, lymphocytosis, eosinophilia and myelocytis is unusually complete. The chapters on hemoglobin, fibrin, lipemia, melanemia and hemorrhage is also full of interesting and instructive data.

Accompanying these chapters are two beautiful full-page colored plates by the author himself, portraying the different varieties of leucocytes, and the malarial parasites, also the leucocytes, lymphocytes, eosinophiles, nucleated red corpuscles, myelocytes such as are seen in leukemia and the polymorpho-nuclear neutrophiles, such as are seen in leucocytosis.

Book II., Part I., is devoted to the diseases of the blood, and chapters follow describing the blood changes in the primary anemias, chlorosis, leukemia and Hodgkin's disease. Two colored plates by the author show beautifully the microscopic appearance of the blood in lymphatic leukemia, with excess of small lymphocytes, and with excess of the large lymphocytes, and Plate IV., showing the varieties of nucleated red cells, as microblasts, megaloblasts, polychromatophilic cells and normal red cells.

Part II. is devoted to acute infectious diseases, as in fevers, pneumonia, typhoid and diphtheria, measles, scarlet fever, variola, cholera, erysipelas, septicemia, grippe, abscess, appendicitis, pelvic inflammation, yellow fever, gonorrhea, glanders, bubonic plague, actinomycosis and trichinosis.

Parts III., IV. and V. are devoted to chronic infectious diseases ; diseases of special organs and malignant diseases, blood parasites and intestinal parasites. Then follows a chapter on the blood in infancy, its general characteristics and the anemias of infancy. These chapters abound in tables, clinical cases and deductions, which can only be appreciated by consulting the book.

The ground gone over by the author can be determined by the very complete bibliography, covering fifty-one pages and embracing the blood literature of all languages.

The work on the whole is a credit to American medicine and should find a place on the table of all progressive physicians. It would be difficult to say wherein it could be improved.

The author is to be congratulated in having William Wood & Company as his publishers, thus insuring the highest excellence in its construction. W. C. K.

